# Enhancing public health infectious disease response through the Australian Pathogen Genomics program (AusPathoGen): evaluation protocol

**DOI:** 10.3389/fpubh.2025.1587112

**Published:** 2025-07-08

**Authors:** Tehzeeb Zulfiqar, Angeline S. Ferdinand, Danielle M. Cribb, James D. H. Ong, Brad Astbury, Kathryn Glass, Son Nghiem, Liliana Bulfone, Nhung Mai, Daisy Wang, Susan Trevenar, Patiyan Andersson, Alireza Zahedi, Martyn David Kirk

**Affiliations:** ^1^Department of Applied Epidemiology, National Centre for Epidemiology and Population Health, College of Health and Medicine, Australian National University, Canberra, ACT, Australia; ^2^Microbiological Diagnostic Unit Public Health Laboratory, The Peter Doherty Institute for Infection and Immunity, Department of Microbiology and Immunology, The University of Melbourne, Melbourne, VIC, Australia; ^3^Centre for Pathogen Genomics, The Peter Doherty Institute for Infection and Immunity, Department of Microbiology and Immunology, The University of Melbourne, Melbourne, VIC, Australia; ^4^Centre for Health Policy, University of Melbourne, Melbourne, VIC, Australia; ^5^Public and Environmental Health, Pathology Queensland, Queensland Health, Brisbane, QLD, Australia

**Keywords:** pathogen genomics, whole genome sequencing, implementation science research, infectious disease, protocol

## Abstract

**Introduction:**

Pathogen genomics is rapidly becoming a cornerstone in the surveillance and response to infectious diseases. However, there is little evidence on how it shapes strategies for effective public health response and decision-making. This paper presents the evaluation protocol for the Australian Pathogen Genomics (AusPathoGen) program, which aims to assess the utility of whole genome sequencing in informing public health responses to infectious diseases in Australia.

**Methods:**

A mixed methods approach will be adopted to systematically explore the utility of whole genome sequencing in public health action and decision-making through a series of linked projects. Methods include situation assessment surveys of Australian public health laboratories, expert elicitation, and case study analysis. The situation assessment surveys will gather data on public health laboratories’ processes, practices, and associated costs for whole genome sequencing. Expert elicitation will seek views on the prioritization of pathogens for whole genome sequencing. Case studies of specific pathogens and outbreaks will serve as the basis for both impact assessment and qualitative comparative analysis. Genomic and epidemiological data will shed light on the influence of whole genome sequencing on outbreak response.

**Discussion:**

This comprehensive evaluation of pathogen whole genome sequencing in Australia will enhance our understanding of how this data can be applied in public health response and decision-making. The methods discussed can be adapted to different public health pathogen genomic surveillance systems globally. Undertaking evaluation of such systems is crucial for identifying areas of improvement and providing recommendations to optimize quality, efficiency and resource allocation of pathogen genomics to improve public health responses.

## 1 Introduction

The advent of pathogen genomic technology has revolutionized our understanding of pathogen transmission dynamics (1). This technology is increasingly being used to identify and characterize infectious agents (pathogens), trace the trajectory and evolution of pathogens, and shape public health strategies and interventions aimed at infectious disease and antimicrobial resistance (AMR) (2–4). In contrast to traditional molecular typing methods (for example, multi-locus sequence typing (MLST) and multi-locus variable-number tandem-repeat analysis (MLVA)), pathogen genomic technology offers a quick and dependable approach to determine the comprehensive genetic makeup of pathogens (5). With pathogen genomics, we can now routinely and economically generate full-length genome sequences in near real-time (4) which provides high resolution of genomic data, enabling distinction of closely related pathogens and supporting surveillance and outbreak investigations.

To ensure an effective public health response, real-time integration of genomic and epidemiological data is critical, as is data sharing within countries and across international borders. In 2010, the United States Food and Drug Administration (FDA) established GenomeTrakr to characterize and detect foodborne outbreaks both nationally and internationally by sharing genomic data among US laboratories and global public health agencies (6, 7). In 2014, Public Health England (PHE) also launched a centralized pathogen genomics service to integrate genomic and epidemiological data for an effective public health response (8). By 2016, 26 European countries reported using pathogen genomics in routine public health surveillance (9, 10). The emergence of coronavirus disease 2019 (COVID-19) led to rapid investment in pathogen genomics initially across multiple high-resource countries to support the public health response to the pandemic. This investment facilitated pathogen identification, monitoring of virus evolution and transmission, identification of variants of concern, and rapid data sharing both within countries and internationally (7, 11). In 2020, AusTrakka was deployed in Australia to facilitate consistent genomic data sharing and reporting between public health laboratories (PHLs) and units (PHUs) across the country to improve national public health surveillance and response during the COVID-19 pandemic (12).

The World Health Organization (WHO) has identified monitoring and evaluation of genomic-informed surveillance systems as a key component of ensuring appropriate implementation and public health benefit from pathogen genomics (11). However, there has been limited evaluation undertaken to assess changes in public health outcomes from the use of pathogen genomics, the utility of pathogen genomics in public health decision-making, or factors that support appropriate use of pathogen genomics data. As such, there is a lack of evidence to identify areas for investment, capacity building and training to support genomics-informed surveillance and outbreak responses. To address this gap, the Pathogen Genomics in Public HeAlth Surveillance Evaluation (PG-PHASE) framework was developed to better understand how pathogen genomics is used in public health via a systems approach (13). The PG-PHASE framework consists of three phases: pathogen genomics laboratory procedures from sample and isolate collection to analysis; reporting and communication of results to end-users; and utility of pathogen genomics data for public health response (13).

In 2020, the Australian Government funded the Australian Pathogen Genomics (AusPathoGen)^1^ program to improve infectious disease responses by integrating pathogen genomics, epidemiological insights, and surveillance data at the population level. One of the aims of AusPathoGen is to evaluate the utility and cost-effectiveness of genomics-based public health responses (14). This evaluation will provide tangible evidence for Australian policymakers on the utility of pathogen genomics in public health and inform future resource allocation to facilitate the integration of genomics into routine public health practice. Such integration is important to improve healthcare services, collect research data and advance diagnostics and treatments (15). In this paper, we describe the methodology of four interconnected studies to achieve this program aim. The present paper mainly focuses on evaluation and implementation studies to assess the public health utility of pathogen genomics in Australia.

The evaluation and implementation studies have been informed by the PG-PHASE framework, which will enable us to assess the application and use of pathogen genomics at various stages of Australia’s public health response (13). This study aims to provide a comprehensive assessment of a pathogen genomics-based public health system, which, to our knowledge, has not been extensively evaluated previously in any country.

## 2 Materials and methods

**Figure 1:**
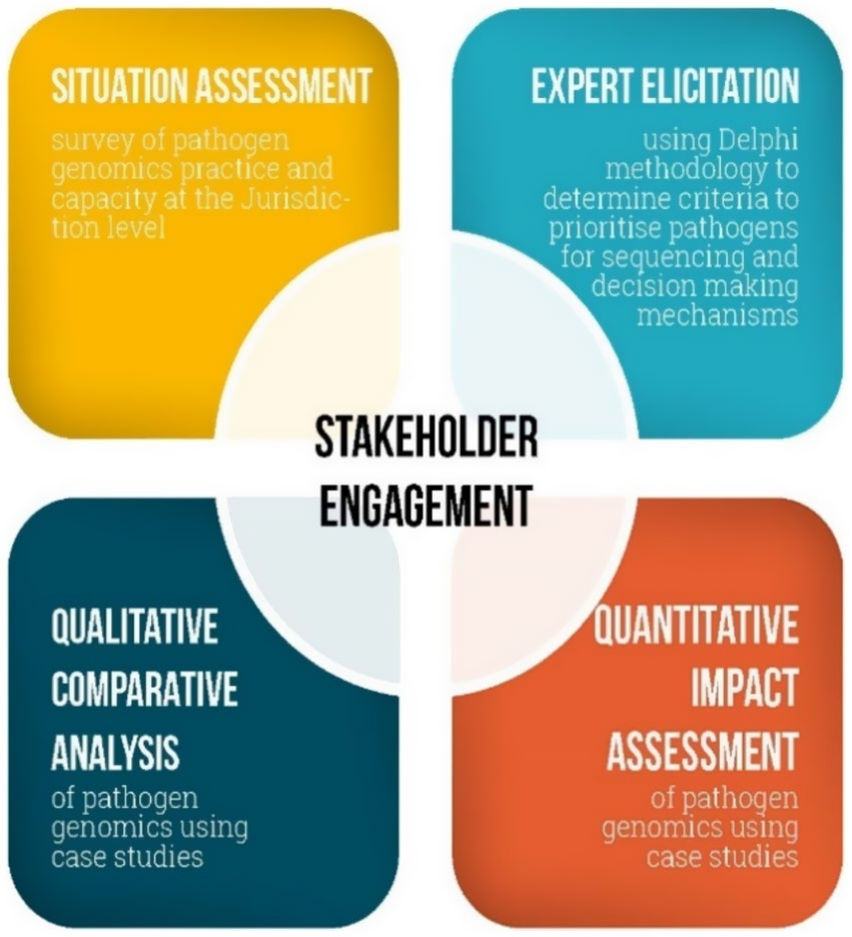
The interconnected projects assessing utility of public health pathogen genomics in Australia.

The AusPathoGen evaluation and implementation aim addresses four research questions that will be addressed through four interconnected mixed-methods projects (Table 1). These projects are designed to provide a whole-of-system understanding of pathogen genomics and contribute to the evidence underpinning implementation of pathogen genomics in public health. The situation assessment will capture current pathogen genomics practice and capacity at the jurisdictional level. In conjunction with an understanding of jurisdictional capacity, the expert elicitation will provide guidance on relevant criteria and mechanisms to determine how to use this capacity to prioritize pathogens for sequencing. Qualitative comparison analysis will be used to identify relevant factors to support the utilization of pathogen genomics data in public health decision-making and outbreak responses, while quantitative impact assessment will determine public health outcomes from the use of pathogen genomics. All projects will be guided and supported by engaging stakeholders at the design, implementation and dissemination phases (Figure 1). In the following sections, we will describe these projects in detail.

**Table 1:** Research questions and research projects underpinning the evaluation.

Research question	Research method
Q1. What is the current pathogen genomics capacity and practice of public health laboratories across different Australian jurisdictions?	Situation assessment surveys: A series of four public health laboratory surveys, designed to provide a snapshot of current public health pathogen genomics practice across Australian jurisdictions.
Q2. What are the criteria and mechanisms that should inform the prioritization of pathogens for sequencing?	Expert elicitation: Using the Delphi methodology to build consensus on criteria to inform prioritization of pathogens for sequencing and mechanisms to make decisions regarding prioritization of pathogens.
Q3. What are the key factors across surveillance and outbreak investigations that contribute to or hinder the use of pathogen genomic data in public health decision making and implementation in Australia and New Zealand?	Qualitative comparison analysis: Systematic examination of various factors influencing the use of pathogen genomics data by end-users to inform infectious disease surveillance and outbreak responses in Australia and New Zealand.
Q4. What is the impact of pathogen genomics on public health outcomes in surveillance and outbreak investigations?	Impact assessment: Quantitative assessment of specific outbreaks to determine the impact of pathogen genomics on public health outcomes.

### 2.1 Situation assessment of pathogen genomics capacity and practice in Australian public health laboratories

Baseline data on the ability of PHLs in Australian jurisdictions to perform pathogen genomics for public health purposes is currently lacking. The objective for the situation assessment is to address this knowledge gap, serving as a reference point for evaluation and identification of areas for improvement.

**Figure 2:**
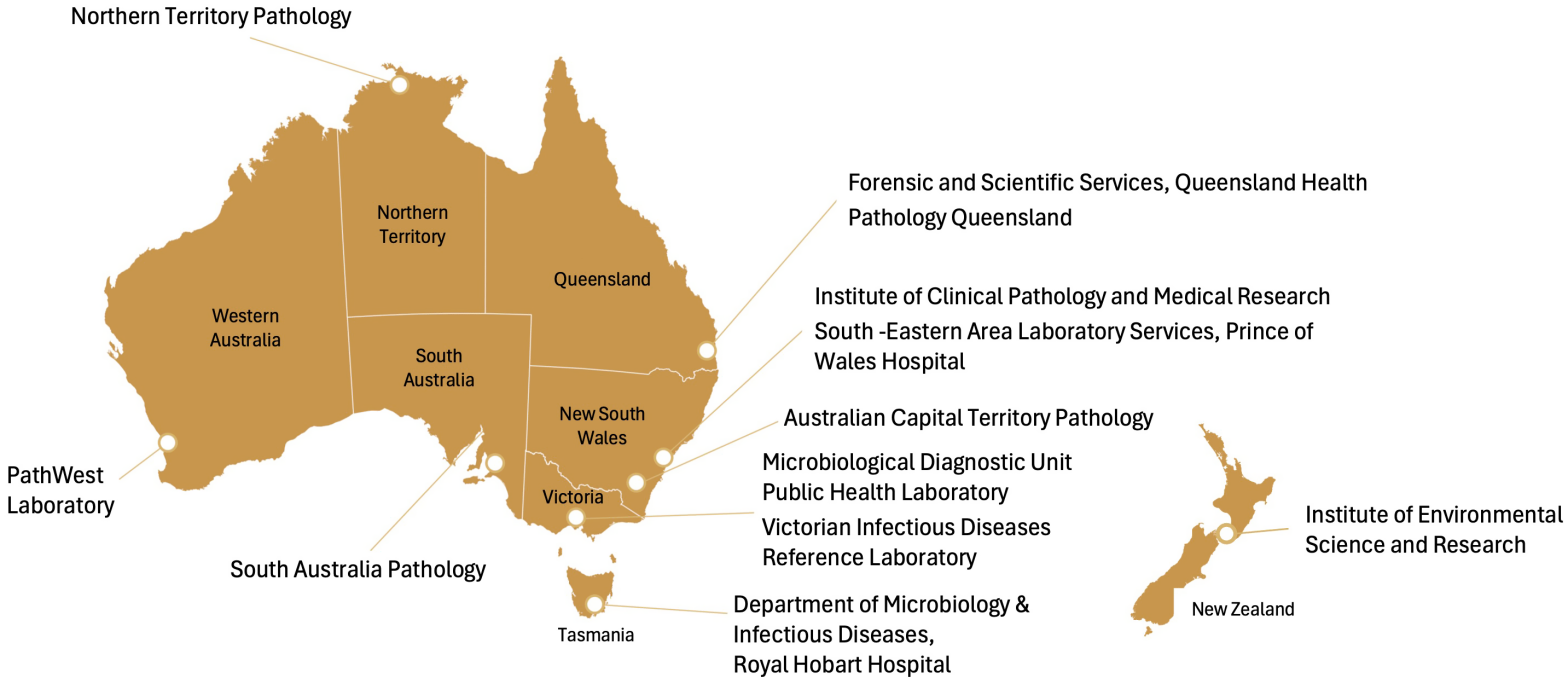
Public health laboratories in Australia. ACT, Australian Capital Territory; SEALS, South Eastern Area Laboratory Services.

Four online surveys, each designed for a separate aspect of laboratory operations directly or indirectly related to pathogen sequencing, will be developed in REDCap 14.6.11 to obtain a snapshot of existing capacity and practices relating to pathogen genomics across Australian jurisdictions. These include functions related to (1) laboratory administration, (2) processing and referral, (3) data analysis and bioinformatics and (4) costing and staffing. Using routinely collected operational data; these surveys will collate comprehensive information from all 11 public health laboratories across Australia (Figure 2).

The respondents for each survey may be different; for example, a respondent to the survey on pathogen genomics laboratory administration may be the director, the chief scientist or senior scientist of the PHL, whereas for the data collection and bioinformatics survey, bioinformaticians, microbiologists or genomic epidemiologists may be the respondents (Table 2).

**Table 2:** Respondents for each evaluation project.

Evaluation project	Respondents of each survey
Situation assessment survey of pathogen genomics capacity and practice in Australian public health laboratories	
Survey on laboratory administration	Laboratory directors Senior scientists Chief scientists
Survey on laboratory data collection and bioinformatics	Bioinformaticians Microbiologists Genomic epidemiologists
Survey on laboratory processing and referrals	Scientists Bioinformaticians
Survey on laboratory costing and Staffing	Laboratory directors Chief scientists Senior scientists Laboratory managers Finance managers
Expert elicitation for prioritizing pathogens for pathogen genomics in Australia	
	Public health experts from infectious disease surveillance and response section of health departments Researchers with pathogen genomics or infectious disease research background Laboratory directors Senior scientists Genomic epidemiologists Microbiologists Bioinformaticians Infectious disease physicians Experts from hospital regulatory bodies Veterinary science and One Health experts
Qualitative comparative analysis to inform infectious disease surveillance and outbreak responses in Australia and New Zealand	
	Genomic epidemiologists Infectious disease physicians Public health experts from infectious disease surveillance and response section of health departments
Impact assessment to determine the impact of pathogen genomics on public health outcomes	
	Genomic epidemiologists Bioinformaticians Laboratory scientists Microbiologists Public health experts from infectious disease surveillance and response section of health departments Infectious disease physicians

The situation assessment questionnaires will be piloted in two PHLs prior to rollout to ensure they are fit for purpose, specifically regarding the availability of requested data, ease of understanding and time to complete the survey.

Data will be exported to Microsoft Excel (V2406) and then to STATA V18 for analysis. The data will be reviewed for completeness and consistency. We will contact laboratories to clarify any uncertainties and request additional information if needed. After data cleaning, we will carry out preliminary descriptive data analysis using STATA V18. The preliminary results of each laboratory will be shared with individual laboratories for validation. At this stage, we will also ask laboratories for additional information if any data are missing or if further clarification is needed. The final report will include aggregated data across all laboratories.

### 2.2 Expert elicitation for prioritizing pathogens for pathogen genomics in Australia

In Australia, the National Notifiable Diseases Surveillance System (NNDSS) monitors over 70 diseases of public health significance (16). However, the resource-intensive nature of pathogen genomics technology necessitates a prioritization strategy for pathogen selection as sequencing every pathogen is not feasible. Uncertainties exist around the mechanisms of selecting pathogens for sequencing, the decision-making process, and the circumstances surrounding pathogen prioritization. The objective for this expert elicitation is to provide evidence toward developing a framework for mechanisms and criteria to prioritize pathogens for sequencing informed by expert views of what needs to be considered when prioritizing pathogens for sequencing, and what pathogens should be sequenced.

Expert elicitation is a structured, mixed-methods process frequently employed to capture expert insights and judgments, especially in areas with limited or uncertain evidence (17) where empirical data may be limited or for addressing questions not suitable for experimental or epidemiological methods (18–20). This method is often employed in fields like science, engineering, and research to inform decision-making processes and model predictions (19). To reach a consensus, the Delphi methodology will be used which involves administering multiple rounds of structured questionnaires to experts, who provide anonymous input and revise feedback from the group (19). This iterative approach aims to reach a convergence of opinions to achieve consensus on a particular topic among a panel of experts (19, 20).

For this expert elicitation, we will develop a survey in REDCap 14.6.11 that will include statements identified from a literature review and those suggested by the research team. The survey will include statements on mechanisms, processes and criteria for determining priority pathogens for sequencing and identify circumstances under which priority pathogens should be sequenced. Each statement in the survey will have an option to provide comments, including reasons for their selected rating, suggestions for alternative statements, and any other general observations. The survey will be piloted on two to four pathogen genomics experts from different laboratories to check the language, terminologies used, flow of the questions, and estimated time needed to complete the survey.

Experts from diverse professional backgrounds (Table 2) across all Australian jurisdictions will be invited to participate in two rounds of expert elicitation. A survey link will be emailed to experts, where they will be asked to rate statements on a 5-point Likert scale and to provide comments and suggestions or reasons of their rating for each statement. We plan to recruit 30 to 40 participants for the study, considering the limited number of experts in the field (21). Each round will be open for 4–6 weeks, with an option to extend the survey timeline if the response rate is low. We will also send weekly reminders to experts to complete the study.

There may be uneven numbers of participants across response groups, given the differing proportions of professions that play a role in the implementation of pathogen genomics. As responses for the survey are received, we will review the balance of expert groups represented by respondents; if there is an underrepresentation of a core group, targeted recruitment of this group will be undertaken.

The survey data will be exported to Microsoft Excel (V2406) and cleaned and analyzed in STATA V18. Qualitative responses in the survey will be managed and analyzed in ATLAS-ti (v.22).

Survey analysis will include descriptive analysis of professional and demographic characteristics of respondents such as location, profession, education, and years of experience. Since there are no established criteria for determining consensus, consensus cut-offs are selected based on common practices found in the literature (19). In both rounds of the survey, statements with 80% or more of respondents agreeing or disagreeing will be considered a consensus, between 60 and 79% agreement or disagreement as partial consensus, and below 60% agreement or disagreement as no consensus.

Qualitative analysis of free text from round one will be used to identify new statements or to modify existing statements based on expert opinion. Participants from round one will receive feedback comparing their responses to overall ratings from other experts. This feedback will include statements that reached full consensus as well as those that reached partial consensus.

In round two, experts will be asked to re-rate statements with partial consensus and evaluate any new or modified statements developed through qualitative analysis of comments from round one.

At the end of round two we will exclude statements from the framework that did not achieve consensus in both rounds. Statements that achieve consensus in either round will inform the framework for prioritizing pathogens for sequencing.

The analysis of qualitative responses or statements and comments provided by the experts in both rounds will be analyzed using thematic analysis. Drawing on both deductive and inductive methods, the analysis will provide context to the ratings, resulting in a more organized, rigorous, and analytically sound method to inform the final framework development (22). We will analyze responses using *a priori* coding based on predetermined aims and objectives to ensure alignment with the research goals. Following this, open coding will be applied to identify any additional concepts and themes that emerge from the data. Data triangulation of both survey and qualitative data will lead to a more robust, credible and meaningful analysis for developing the framework to describe mechanisms and criteria of pathogen prioritization for pathogen genomics (22).

### 2.3 Qualitative comparative analysis (QCA)

End users such as policymakers and public health authorities are increasingly relying on pathogen genomic data for surveillance and outbreak responses against infectious diseases (10, 13, 23). The utilization of this data depends on several factors, including timeliness of reports (23), ease of interpreting pathogen genomic data (24) and end-users’ genomic literacy (25). However, there is a lack of evidence identifying key factors that influence the use of this data by end-users to inform surveillance and outbreak responses. To fill this research gap, we will conduct a QCA of various case studies from Australia and New Zealand (26). QCA is a case-oriented methodology that compares cases or units of analysis to identify key factors affecting the probability of an outcome occurring (27). For this study, cases describe specific infectious disease surveillance programs or outbreak responses where pathogen genomic data was available.

A literature review will be conducted to generate a rubric containing a definition and scoring criteria for each outcome and contextual factor. Cases will be purposively selected to reflect a range of different outcomes, with variations in the presence or absence of individual factors among cases. Utilizing a mixed methods approach, we will systematically compare and analyze cases where pathogen genomics has been used in public health systems across Australia and New Zealand.

Data for these cases will be collected from reviewing publicly available and internal documents such as program guidelines, policy papers, procedure manuals, and genomic data reports. Semi-structured key informant interviews will also be conducted with technicians and end users to gain in-depth knowledge of each case. The interview questions for each case study will focus on the extent that pathogen genomic data were used, the perceived impact of pathogen genomics on the public health response, and the factors influencing the use of pathogen genomic data. Sensitive data from internal documents and interviews will be stored in accordance with procedures outlined in the ethics application; that is, this information will be held in secure, password-protected University systems accessible only by the named investigators. Information from each case will be summarized in a case report. Respondents will have the opportunity to review the case report for accuracy and to ensure confidentiality is appropriately maintained.

The case reports and rubric will inform how each case will be scored on each outcome and factor. These scores will be placed in a data matrix which will be converted to a truth table listing all possible factor combinations. The truth table will then be analyzed via Boolean minimisation to generate a set of pathways that independently lead to the outcome. These pathways will be interpreted alongside the cases to provide rich explanations of how pathogen genomic data is used in public health. This analysis will uncover various pathways leading to the use of pathogen genomic data in decision-making and policy development. As such, reporting will focus on the analyzed factors, rather than the specifics of the individual case studies. The respondents for this study will be individuals who process or present pathogen genomic data (Table 2). Given an inclusion of 10–15 cases and a diversity of respondents for each case, the estimated number of respondents is 50–75. The detailed protocol of QCA study is published separately (26). This project will provide explicit targets for investment and surveillance system strengthening to support more effective use of pathogen genomic data in infectious disease response.

### 2.4 Quantifying the impact of pathogen genomics on public health outcomes

To assess the quantitative impact of pathogen genomics in outbreak responses, we will identify pathogens for case studies in consultation with stakeholders including genomic epidemiologists, bioinformaticians, laboratory scientists, microbiologists, and public health clinicians. Outbreaks where the use of pathogen genomic data has been particularly impactful will be selected to further understand the circumstances under which pathogen genomics is most useful.

Epidemiological and genomic data will be analyzed, guided by the PG-PHASE evaluation framework. This framework provides examples of relevant indicators for pathogens such as *Listeria monocytogenes*, *Mycobacterium tuberculosis* and SARS-CoV-2 (13).

The quantitative data will be cleaned and analyzed in STATA V18. The analysis will focus on understanding the contributions of genomic data in identifying transmission pathways and contamination sources and informing public health responses. Specific indicators of these comparisons will vary according to pathogen but may include epidemiological measures such as the number of identified cases; size, duration, and number of identified clusters; the proportion of cases linked to clusters; and the proportion of cases or clusters traced to a contamination source. Relevant economic data will also be collected where possible. Economic indicators may include resource allocation for epidemiological investigations and infection control investigations and actions; direct health care costs; financial losses relating to foodborne disease outbreaks (e.g., food recalls); clean-up costs in relation to water and environmental outbreaks; and costs associated with sick leave, both for employers in the form of reduced productivity and for employees as loss of income. This project will provide a stronger understanding of the circumstances under which public health pathogen genomics is most impactful and, in combination with the QCA project, enable more targeted investment of resources.

### 2.5 Stakeholder engagement

Stakeholder engagement is central to the design and implementation of the evaluation and will be carried out regularly during the evaluation process. To ensure diversity and inclusion, we will engage a broad and representative group of stakeholders who are either responsible for producing pathogen genomic data or are end users of these data. These stakeholders will have backgrounds in infection prevention, surveillance and control, and pathogen genomics. Stakeholders will include microbiologists, epidemiologists, genomic epidemiologists, infectious disease clinicians, academics, and public health registrars. These professionals will be invited from public health laboratories, public health units, clinics, and hospitals across all Australian jurisdictions.

Additionally, we will invite representatives from various organizations, including the Australian Department of Health and Aged Care, Department of Agriculture, Fisheries and Forestry, OzFoodNet, Food Standards Australia New Zealand, Communicable Disease Genomic Network, and the Public Health Laboratory Network. Academics focused on pathogen genomics, along with infection prevention and control will be integral members of the stakeholder group.

To ensure comprehensive stakeholder engagement, we will implement systematic feedback mechanisms to collect and integrate stakeholder input into the project. This will include engaging stakeholders in quarterly project reference group meetings to provide guidance on operational issues and evaluation activities. Stakeholders will also be invited to identify potential respondents for research activities, review study tools such as questionnaires or draft reports, and identify case studies for research. Additionally, they will assist in addressing implementation challenges, facilitating communication, and helping disseminate the results of the evaluation.

Regular updates will be shared through quarterly newsletters. Annual seminars will be held for the broader AusPathoGen project, featuring representation from implementors and stakeholders across all project elements. These seminars will provide opportunities to present interim findings, gather feedback, and facilitate in-depth discussions and collaboration among stakeholders.

## 3 Discussion

This protocol paper outlines a comprehensive whole-of-system evaluation of the public health utility of pathogen genomics in Australia. Collectively, the four projects planned for this evaluation will provide a picture of current pathogen genomic practices in Australia and New Zealand to inform decisions regarding which pathogens should be prioritized for sequencing and under what conditions. They will also provide an understanding of the benefits of public health pathogen genomic, programs identify areas of improvement and provide recommendations to guide a sustainable and effective pathogen genomic surveillance system. The evaluation approach, consisting of four interlinked mixed methods projects, is in line with WHO guidance on monitoring and evaluation of genomics-informed surveillance systems. This guidance highlights the importance of landscape analyses such as situation assessments and case studies to understand both quantitative and qualitative aspects of implementation (28).

There is a lack of implementation science studies investigating the utility of pathogen genomics from a public health system perspective. Current evidence primarily focuses on the technical capacity of public health laboratories to conduct genomics for public health surveillance (10, 29), and the challenges in transitioning from proof-of-concept studies to routine application of pathogen genomics. These challenges include data integration, quality of contextual data, sampling strategies, meaningful interpretation of results during outbreaks and surveillance activities (30), sustainable funding and resources (31), and turnaround time for genomic data to be available for public health action (23). Evidence is also available on the reporting and interpretation of genomic reports by end-users (24, 31), end-users’ views of implementing pathogen genomics in their work activities relating to infectious disease surveillance and control (17, 25) and health professionals’ readiness and needs for pathogen genomics in their clinical practice (32). Results from these studies show that pathogen genomics is more effective for infection prevention and control compared to traditional typing methods as it provides higher resolution and accuracy to better understand infectious diseases and antimicrobial resistance patterns (4, 5). However, common barriers for genomics-informed surveillance include issues with specimen access, data flow and sharing, turnaround times, computing infrastructure, cost, expertise, and training (10, 25, 31, 32).

### 3.1 Strengths and limitations

Using four interlinked projects provides a whole-of-system overview of public health pathogen genomics in Australia, while also enabling targeted examination of factors contributing to utilization of pathogen genomic data. The positioning of the evaluation projects within the broader AusPathoGen program will allow for stronger connections with potential respondents across the public health pathogen genomics surveillance system, in particular public health laboratories and public health authorities and end users of genomic data.

As with any large project, there are some foreseeable and unforeseeable challenges. Some potential challenges common to all projects in the evaluation include time constraints to complete all activities, survey fatigue, scheduling difficulties, confidentiality concerns and data quality (33). Due to the small number of stakeholders working on pathogen genomics and infection prevention and control in Australia, we may encounter one or more of these challenges, which may affect the quality and reliability of the information. However, as most of our respondents are key stakeholders in the research, we anticipate high interest and engagement towards the evaluation projects. We will provide respondents ample time to complete surveys and send multiple reminders in case of missing responses. We will also be flexible in scheduling interviews and assure our respondents of maintaining confidentiality.

## 4 Conclusion

This comprehensive evaluation of pathogen whole genome sequencing in Australia will enhance our understanding of how this data can be applied in public health response and decision-making. While the current projects were developed for the Australian context, the approaches used have been underpinned by frameworks designed to be broadly applicable. As such, the methods discussed are flexible and able to be adapted to different public health pathogen genomic surveillance systems globally. Undertaking evaluation of such systems is crucial for identifying areas of improvement and providing recommendations to optimize quality, efficiency and resource allocation of pathogen genomics to improve public health responses. Adaptation of these evaluation methods or application of the underlying evaluation frameworks may be undertaken individually or with support from the AusPathoGen evaluation team.

## Data Availability

The original contributions presented in the study are included in the article/supplementary material. Further inquiries can be directed to the corresponding author.

## Footnotes

^1^ https://www.auspathogen.org.au/

## Author’s note

Findings from the evaluation projects will be presented at project reference group meetings, AusPathoGen executive group meetings, AusPathoGen symposiums, and national and international conferences. Key results will also be published on the AusPathoGen website and in quarterly newsletters. Additionally, project results will be published in peer-reviewed academic journals to ensure wider dissemination to implementors and stakeholders globally.

## Group members of the AusPathoGen Program

For information on the AusPathoGen program, please visit https://www.auspathogen.org.au.
